# Exploitation of Mangrove Endophytic Fungi for Infectious Disease Drug Discovery

**DOI:** 10.3390/md16100376

**Published:** 2018-10-10

**Authors:** Danielle H. Demers, Matthew A. Knestrick, Renee Fleeman, Rahmy Tawfik, Ala Azhari, Ashley Souza, Brian Vesely, Mandy Netherton, Rashmi Gupta, Beatrice L. Colon, Christopher A. Rice, Mario A. Rodríguez-Pérez, Kyle H. Rohde, Dennis E. Kyle, Lindsey N. Shaw, Bill J. Baker

**Affiliations:** 1 Department of Chemistry, University of South Florida, Tampa, FL 33620, USA; dhdemers@mail.usf.edu (D.H.D.); m.a.knestrick@gmail.com (M.A.K.); 2 Department of Cell Biology, Microbiology and Molecular Biology, University of South Florida, Tampa, FL 33620, USA; rfleeman@utexas.edu (R.F.); rtawfik@mail.usf.edu (R.T.); shaw@usf.edu (L.N.S.); 3 Department of Global Health, University of South Florida, Tampa, FL 33613, USA; aazhari@kau.edu.sa (A.A.); asouza@taskforce.org (A.S.); bvesely@health.usf.edu (B.V.); Christopher.Rice@uga.edu (C.A.R.); DENNIS.KYLE@uga.edu (D.E.K.); 4 Division of Immunity and Pathogenesis, Burnett School of Biomedical Sciences, University of Central Florida, Orlando, FL 32827, USA; Mandy.Netherton@ucf.edu (M.N.); Rashmi.Gupta@ucf.edu (R.G.); Kyle.Rohde@ucf.edu (K.H.R.); 5 Department of Molecular Medicine, University of South Florida, Tampa, FL 33613, USA; Beatrice.Colon@uga.edu; 6 Instituto Politécnico Nacional, Centro de Biotecnología Genómica, Blvd. del Maestro esq. Elías Piña s/n. Reynosa 88710, Tamaulipas, Mexico; mrodriguez@ipn.mx

**Keywords:** endophytic fungi, epigenetic modification, mangroves, screening, infectious disease drug discovery

## Abstract

There is an acute need for new and effective agents to treat infectious diseases. We conducted a screening program to assess the potential of mangrove-derived endophytic fungi as a source of new antibiotics. Fungi cultured in the presence and absence of small molecule epigenetic modulators were screened against *Mycobacterium tuberculosis* and the ESKAPE panel of bacterial pathogens, as well as two eukaryotic infective agents, *Leishmania donovani* and *Naegleria fowleri*. By comparison of bioactivity data among treatments and targets, trends became evident, such as the result that more than 60% of active extracts were revealed to be selective to a single target. Validating the technique of using small molecules to dysregulate secondary metabolite production pathways, nearly half (44%) of those fungi producing active extracts only did so following histone deacetylase inhibitory (HDACi) or DNA methyltransferase inhibitory (DNMTi) treatment.

## 1. Introduction

A growing body of research on drug resistance among pathogens [1,2,3,4] and previously neglected or poorly understood infectious diseases [5,6,7,8,9] highlights the persistent need for efficient drug discovery efforts in nearly all infectious disease areas. Marine natural products have, historically, been important in the development of therapeutics for infectious diseases, and contemporary efforts in these areas continue to prove their value [10,11,12,13,14,15,16]. Herein, we report the combined results of screening a library of marine fungal extracts against: *Enterococcus faecium*, *Staphylococcus aureus, Klebsiella pneumoniae, Acinetobacter baumannii, Pseudomonas aeruginosa, Enterobacter cloacae*, (i.e., the ESKAPE pathogens), *Mycobacterium tuberculosis*, *Leishmania donovani*, and *Naegleria fowleri*. These diverse microbial targets represent some of the most important threats to human health today.

While invertebrates remain a major source of new bioactive marine compounds, developments in microbial isolation, culture, and genome-sequencing techniques continue to expand the natural products frontier of marine microbial sources [13,17,18,19,20,21,22,23,24,25,26]. Mangrove forests are marine-margin habitats regarded for their microbial, chemical, and biological diversity [27,28,29,30,31]. While endophytic microorganisms are well studied in South-East Asian mangroves [27,28,30], the microbial inhabitants of the expansive mangrove forests in the Americas and the Caribbean remain largely unexplored. Surrounded by these “new world” mangroves in North America, our microbial library consists primarily of fungal isolates from mangrove and mangrove associated trees including: *Rhizophora mangle*, *Avicennia germinans*, *Laguncularia racemose*, *Conocarpus erectus*, and *Coccoloba uvifera*.

**Figure 1:**
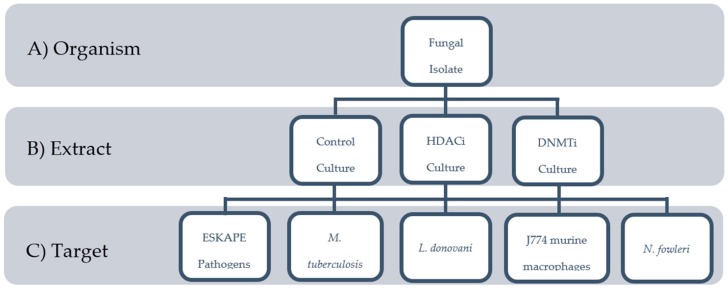
Structure of the screening campaign. Each fungal isolate was (**A**) grown under 3 culture conditions, (**B**) generating 3 extracts per organism. Each extract was (**C**) screened in 5 assay systems. The screening of 530 isolates resulted in the production of 1608 extracts, and comprehensive screening in multiple assays resulted in a total of 8040 data points.

Generally, endophyte isolation protocols are designed to target as many genera of fungi as possible. Nevertheless, because fungi are capable of regulating their biosynthetic pathways in response to variations in both natural and artificial growth conditions [32,33,34], many fermentation techniques have been developed to induce the production of previously undetected, bioactive chemistry in fungi [35,36,37,38,39,40,41,42,43,44]. Most strategies, like co-culture or genome mining, focus on a single or small number of isolates or therapeutic targets. While these techniques are quite effective at exploiting the entire biosynthetic potential of an organism, they do not translate particularly well into larger-scale screening campaigns targeting multiple pathogens. To that end, we describe here methodology utilizing small molecule regulation of fungal transcription to enhance extract libraries for the expansion of screening capabilities (Figure 1).

In this study, we employed miniaturized culture conditions [30] and the use of histone deacetylase inhibitors (HDACi) and DNA methyltransferase inhibitors (DNMTi) to examine the effect of epigenetic regulators known to modulate secondary metabolite expression [32,33,34,35,36,37,38,39,40,41,42,43,44,45]. From 530 fungal isolates, 1608 extracts were generated and screened against a panel of infectious disease targets (Figure 1). Potency and cytotoxicity data were used to prioritize lead extracts, defined as those extracts of high interest which would be advanced to scale up and chemical analysis. Comparison among screening results and culture treatments has identified noteworthy trends. The data showcases the diversity and specificity of bioactive natural product extracts from North American mangrove endophytes and supports the use of epigenetic modification as a screening tool.

## 2. Results

### 2.1. Biological Materials

**Figure 2:**
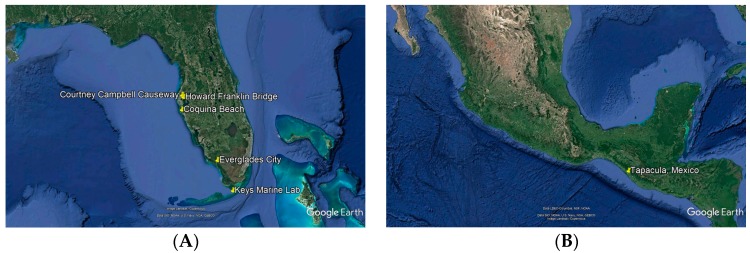
Geographic distribution of sampling sites in (**A**) Clearwater, Sarasota, Everglades City and Layton, Florida; and (**B**) Tapachula, Chiapas, Mexico.

Mangrove tissues (roots, stems, leaves, flowers) were sampled primarily from shoreline communities (Figure 2) in Florida (Courtney Campbell Causeway, Tampa, FL (CC); Coquina Beach, Sarasota, FL (CQ); Everglades City, FL (EG); Howard Franklin Causeway, Tampa, FL (HF); Keys Marine Lab, Layton, FL (KML)) and Mexico (Tapachula, MX (TAP)), with contributions from opportunistic collections in other environments. Surface sterilized plant tissues placed on nutrient agar produced emergent hyphae that were clipped and purified through repeated streaking, yielding approximately 3000 endophytic fungal strains [45,46,47]. A selection of 530 strains were randomly chosen for these screening studies.

### 2.2. Extract Library

The selected fungal strains were cultivated with and without modulators of epigenetic regulation, resulting in 530 extracts each for sodium butyrate (HDACi) treated, 5-azacytidine (DNMTi) treated and non-treated cultures (1608 total cultures) that were extracted with ethyl acetate and distributed into 17 96-well plates at 5 mg/mL in DMSO.

### 2.3. Screening

#### 2.3.1. ESKAPE Pathogens

One or more of the six bacterial pathogens that constitute the ESKAPE panel of bacterial pathogens were sensitive to 203 total fungal extracts. Using serial dilutions of extracts of 200, 100, 50, 25, 10 and 5 µg/mL, scaled scoring (SS) [46] of minimal inhibitory concentrations (MICs) identified the most promising lead extracts. Bioactivity levels above SS 9 were chosen as extracts of interest. Filtering to remove cytotoxic extracts (J774 IC_50_ < 5 µg/mL) produced a collection (Table S1) of 46 *lead extracts* (2.9% of all extracts tested), 24% of which had been cultured under control conditions, 37% under DNMTi conditions, and 39% under HDACi conditions.

#### 2.3.2. *Mycobacterium tuberculosis*

*Mycobacterium tuberculosis* was scored as percent growth inhibition (GI). Sensitivity measured as GI_50_ resulted in 540 fungal extracts with activity at 100 µg/mL. Filtering extracts to select for low cytotoxicity (J774 IC_50_ > 5 µg/mL) and GI_95_ resulted in 100 (6.2% of all extracts tested) extracts as *lead extracts*, including 31% untreated extracts, 33% treated under DNMTi conditions, and 36% treated under HDACi conditions.

#### 2.3.3. *Leishmania donovani*

The 50% inhibitory concentration (IC_50_) for J774 macrophages infected with *L. donovani* found 562 extracts active at 10 µg/mL or less. Filtering the data to select for low cytotoxic extracts (J774 IC_50_ > 5 µg/mL) and high potency (*L. donovani* IC_50_ < 1.0 µg/mL) reduced the *lead extract* set to 116 extracts (7.2% of all extracts), including 41 extracts with no epigenetic modulators (35%), 40 (34%) and 35 (30%) extracts subject to DNMTi and HDACi, respectively (Table S3).

#### 2.3.4. *Naegleria fowleri*

In the assay for *N. fowleri*, 34 extracts displayed >33% inhibition of the amoeba (Table S4) at, on average, 50 µg/mL, 2 of which were deprioritized due to high cytotoxicity (IC_50_ < 5 µg/mL), leaving 32 total *lead extracts* (2% of all extracts). Parsing the screening results by activity level reveals one extract with the indicated >33% inhibition achieved at 5 µg/mL, and two extracts achieving >67% inhibition at 50 µg/mL (Table S4), providing a clear pathway to prioritization of the *N. fowleri* lead extracts. Over half of the extracts active against *N. fowleri* were non-treated (56%), one quarter were HDACi treated, and the remainder were DNMTi treated.

### 2.4. Overall Data Analysis

**Figure 3:**
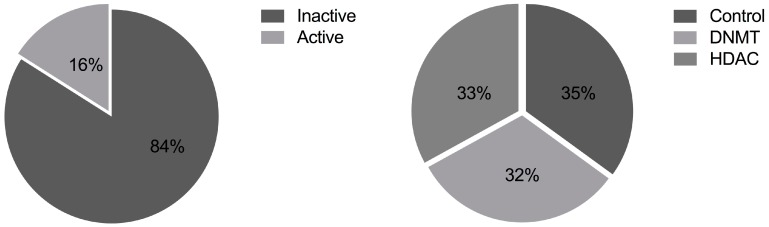
Distribution of (**A**) lead extracts and (**B**) treatment method for lead extracts.

Of the 1608 extracts screened, 254 (16%) were determined to be active (“lead extracts”) in one or more assay (Table S5). These 254 active extracts resulted from 162 endophytes (Table S6), and 72 of these fungi (44%) produced active extracts only when cultured in the HDACi or DNMTi conditions (Figure 3). Specifically, 29 fungi were only active after HDACi treatment, 24 were only active after DNMTi treatment, 19 were active in both HDACi and DNMTi treatments (but not the control), 40 fungi were active only in the control, and 69 fungi were active in the control and at least 1 treatment condition.

**Figure 4:**
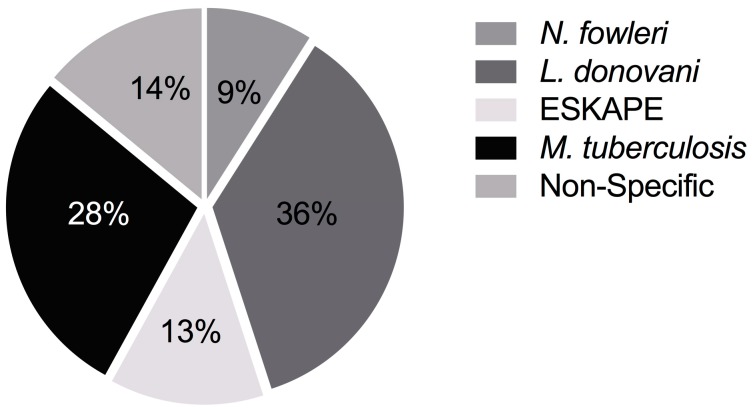
Distribution of selectively active lead extracts in the screening subset, by target.

Only 36 lead extracts (14% of total active, 2% of total screened) showed activity against multiple targets (Figure 4, Table S5). Specific activity against *N. fowleri* was observed in 24 extracts (9%, 1%), 33 extracts (13%, 2%) were active only in the ESKAPE pathogen screen, 71 (28%, 4%) were specific to *M. tuberculosis*, 92 (36%, 6%) were active only against the *L. donovani* infected macrophage.

## 3. Discussion

Endophytic fungi were isolated from mangrove tissues of five species of mangrove or mangrove associated trees from five North American regions (Figure 2), yielding an average of 500 fungal strains from each geographic location. Strains cultured in a manner to enhance epigenetic expression of secondary metabolites were demonstrated to produce broad yet selective bioactivity profiles. Lead extracts, defined as extracts displaying sufficiently high potency (variable among assays) and low mammalian cytotoxicity (>5 µg/mL) to be considered candidates for chemical analysis, were found in 16% of all tested samples (Figure 3). Among individual screens, the overall lead extract hit rate was found to be 2–7%, reflecting the stringent potency and cytotoxicity criteria selected for each assay as guidance for advancing samples to chemical analysis.

Among the bacterial pathogens, *Staphylococcus aureus* was the most sensitive of the ESKAPE panel (Figure S1) to the fungal extracts. While nearly 13% of extracts displayed activity in one or more of the ESKAPE pathogens, only 2.9% of extracts were sufficiently activity (scaled score = 9) [46] to advance as lead extracts. *Mycobacterium tuberculosis* on the other hand proved highly sensitive, with 34% of extracts exhibiting GI_50_ ≤ 100 µg/mL. Focusing on the most promising *M. tuberculosis* activities by restricting the activity to the GI_95_ still produces 100 lead extracts (6.2% hit rate), an encouraging result that holds promise for the discovery of new anti-tuberculosis scaffolds. The pressing need to overcome antibiotic resistance will be advanced by the discovery of new antibiotics with new mechanisms of action [48].

The eukaryotic pathogens studied in this project were the protists responsible for leishmaniasis, *Leishmania donovani*, and primary amoebic meningoencephalitis (PAM), *Naegleria fowleri*, two rare and largely neglected diseases which nonetheless carry a significant disease burden, not merely due to morbidity and mortality [49,50,51], but for social and economic [52] impacts. *L. donovani* was the most sensitive test organism in our project, inhibited at low dose (<1 µg/mL) with low mammalian cytotoxicity (Table S3) by 116 extracts (7.2% of all extracts). Employing a typical natural product molecular mass of 500 g/mol indicates that nearly half of those lead extracts will harbor sub-micromolar compound(s). In contrast, *N. fowleri* was the least sensitive pathogen to our extract set, responding to only 2% of 1608 extracts with low sensitivity (generally 33–66% inhibition at 50 µg/mL, see Table S4).

Using inhibitors of two epigenetic regulatory mechanisms proved profitable in enhancing screening results. Fungi treated with the HDAC inhibitor sodium butyrate and the DNMT inhibitor 5-azacytidine produced extracts that acted as independent screening samples, displaying unique bioactivity profiles from one another and from untreated extracts. This strategy effectively generated an extract library that was functionally three times the size of the microbial isolate library (Figure 2B). Additionally, the data indicates that this methodology successfully accessed otherwise hidden biosynthetic potential, with 44% of active fungi producing activity only in the presence of epigenetic modification. As expected, instances in which the small molecule pressure eliminated or had no effect on activity were also observed (e.g., extracts that were active only in the control, or in both the control and modified conditions), further validating the approach.

A high level of selectivity was observed among the active extracts. Large natural product extract libraries—specifically those of fungal origin—are often considered to be plagued with indiscriminately active nuisance compounds. Nevertheless, these results display a high level of extract selectivity and a hit rate of ~5% in each assay. These statistics strengthen the argument for bioprospecting within the microbial world found in the North American mangrove forests.

It is notable that these conclusions have been generated without chemical analysis of the extracts (HRMS, NMR). With nothing more than a diverse set of bioassay data, these extracts can now be rationally prioritized according to potency, specificity, or modification efficacy. In certain bioassays, further information may be extrapolated; e.g., in the ESKAPE panel, extracts acting against both Gram positive and Gram negative pathogens as compared to those displaying selective activity towards Gram negatives. In smaller extract subsets like this one, this may be sufficient to transition directly into scale-up and structure isolation schemes. For larger extract libraries, or in the search for new and novel chemistry, this front-end bioassay data can inform a more cost and time efficient transition into extract chemical analysis such as HRMS- or NMR-based networking for dereplication efforts. Whatever the next step, the accumulation of seemingly unrelated biological data on an extract library can direct a more efficient, effective compound discovery pipeline.

Scale up and chemical analysis of active extracts identified herein, including the isolation of bioactive compounds, is underway. In the case of rare and neglected disease targets, those with newly developed drug targets, and in the face of growing drug resistance, both new and previously isolated natural products, may be of interest. We believe that this bioassay-driven approach is ideally suited for target specific isolation and investigation of both new and known bioactive natural products for meaningful drug discovery efforts.

## 4. Materials and Methods

### 4.1. Fungal Isolation

Tissues from mangroves (*Rhizophora mangle, Avicennia germinans*, and *Laguncularia racemosa*), associated trees (*Conocarpus erectus*, and *Coccoloba uvifera*), sediments, and marine invertebrates were collected over the course of several years (2010–2014) at sites around Tampa Bay, the Florida Keys, the Gulf of Mexico and Tapachula, Mexico. As previously reported [31], small pieces of organic material were surfaced sterilized in bleach and/or isopropyl alcohol and pressed against or transferred onto various solid agar media types meant to target a large scope of bacteria and fungi. Media preparation was as follows: a nutrient medium (e.g., potato dextrose, Sabaurad dextrose, actino, malt, tryptic soy, or glycerol) was combined with agar, salt, and a combination of antibacterial or antifungal small molecules (e.g., nystatin, cycloheximide, or chloramphenicol). Six to 10 variations on these solid media types were utilized in each collection. Collected tissues were plated in triplicate on each of the media types. During incubation at room temperature, plates were routinely checked for growth and colonies were transferred to isolation plates of similar media composition. Once a pure colony had been established, small subsamples of mycelium were inoculated into a 20% glycerol/water solution for long-term preservation.

### 4.2. Miniaturized Culture Conditions and Extraction Procedures

Generally following methods previously reported [30,31], for each fungal isolate, two 1 cm^2^ pieces of fungal material on agar were inoculated into 1.25 mL of each: untreated SDB (Sabouraud dextrose broth), 100 µM sodium butyrate in SDB, and 100 µM 5-azacytadine in SDB and agitated. Each of these aliquots was poured over 3 g autoclaved brown rice in a 20 mL scintillation vial. Cultures were fermented at 28 °C for 21 days.

Following fermentation, the fungal material was sprayed with approximately 500 µL MeOH and then soaked with 10 mL EtOAc. The cultures were extracted for 24 h, after which time the extract was decanted, dried, and re-solvated to a concentration of 5 mg/mL in DMSO. Samples were transferred into 96-well plates using a TECAN liquid handler. Plates were distributed for bioassay and remaining extracts archived for use in future analysis in deep 96-well plates at −18 °C. 

### 4.3. ESKAPE Bacterial Strains, Growth Conditions, and Microtiter MIC Determination Assays

The ESKAPE pathogen clinical isolates used in this study were obtained from Moffitt Cancer Center and Tampa General Hospital. Overnight cultures were grown in lysogeny broth (LB) or tryptic soy broth (TSB), as described previously [46].

MIC assays were performed as previously described [46]. Briefly, overnight culture of all strains were grown in tryptic soy broth then diluted 1:1000 in cation adjusted Mueller Hinton broth (CA MHB, Difco laboratories, a subsidiary of Becton, Dickinson and Company, Sparks, MD 21152 USA) for the assay. The initial testing started at 200 µg/mL extract concentration in a 96-well microtiter plate and allowed to grow 20 h at 37 °C. The extracts were screened using a tiered MIC approach, with active samples at 200 µg/mL progressed to testing at 100 µg/mL. This approach was used to continue testing to 50, 25, 10, and 5 µg/mL.

### 4.4. Analysis of Antimicrobial Activity against Replicating Mycobacterium tuberculosis

Activity of the fungal compounds was examined against *Mycobacterium tuberculosis* using a reporter-based whole cell assay. The strain *Mtb*-RG, which expresses mCherry and GFP constitutively from the *smyc* promoter and *hsp60* promoter, respectively, was grown to a logarithmic phase (OD_600_ of 0.4–0.8) in 7H9 broth supplemented with OADC (oleic acid, albumin, dextrose, catalase) and kanamycin 50 µg/mL. The culture was diluted to an OD_600_ of 0.05 and added to a black solid bottom 384-well plate containing the fungal compounds at a final concentration of 100 µg/mL to a total volume of 30 µL per well. Rifampicin 10 µM (positive) and 2% DMSO (negative, no drug) controls were also included in the screening plate. The plate was incubated at 37 °C, 5% CO_2_ and fluorescence (excitation/emission 575/485 nm) was measured after 72 h using a Biotek Synergy H4 plate reader (Winooski, VT, USA). The activity of the fungal compounds was calculated as percent inhibition which was determined relative to the no drug control and inhibition by rifampicin taken as 100% using the formula {((DMSO signal-sample signal)/DMSO signal × 100) × 100/Rif inhibition} [15].

### 4.5. Leishmania donovani-Infected Macrophage (IM) Assay

Two thousand J774.A1 cells were seeded in each well of Perkin Elmer CellCarrier-384 Black Optically Clear Bottom plates (Perkin Elmer, Waltham, MA, USA). *Leishmania donovani* amastigotes were centrifuged at 3000 RPM for 5 m and brought up in macrophage media and added to macrophage well at a ratio of 10:1. They were then incubated for 4 h at 37 °C and 5% CO_2_. Non-phagocytized amastigotes were washed away and fresh macrophage media was added and allowed to incubate overnight. Media was removed and extracts diluted in media were added to the 384 well plates and incubated for 72 h. The extracts were all in six concentrations diluted 1:2 starting at 10 μM with positive and negative control and miltefosine standard drug control. The media was removed and fixed in 2% paraformaldehyde (Alfa Aesar, Ward Hill, MA, USA) in media for 15 m. The paraformaldehyde was removed and cells were stained with 5 μM Draq5 (Thermo Scientific, Waltham, MA, USA) diluted in PBS for 5 m. The stain was removed and fresh media was added to the plate. The Operetta (Perkin Elmer, Waltham, MA, USA) high content imager was used to capture six images from the middle of each well using a Far Red filter using 100 ms at 100% excitation at the plane of −1.4 µm. An algorithm in Harmony (Version 4.1, Perkin Elmer, Waltham, MA, USA) software in image analysis mode was programed to find the macrophage nuclei, cytoplasm, and amastigotes within the cytoplasm. The software counted the number of amastigotes per 500 macrophages in each well and calculated IC_50_ values based on non-linear regression of dose response curves.

### 4.6. Cytotoxicity of Mammalian Cell Line

The viability of J774.A1 macrophages was determined by the Cell Titer 96 Aqueous Assay (Promega, Madison, WI, USA) that employs a tetrazolium compound [3-(4,5-dimethylthiazol-2-yl)-5(3-carboxymethoxyphenyl)-2-(4-sulphophenyl)-2H-tetrazolium (MTS)] and electron-coupling reagent, phenazine methosulphate (PMS). Test extracts were serially diluted in 100 μL of media in 96 cell plates using a Biomeck 3000 (Beckman Coulter, Miami, FL, USA). From each well, 10 μL was transferred to another 96 well plate and then received 90 μL J774.A1 in media. The J774.A1 macrophages were in a concentration to have 50,000 cells per well. After 72 h 20 μL of MTS solution was added to each well in the 96 well plates. The plates were then incubated 37 °C and 5% CO_2_ for 4 h to achieve optimal color development. After 4 h of incubation, the OD (optical density) values were determined at 490 nm using a Spectra Max M2 (Molecular Devices, Sunnyvale, CA, USA). The results were presented as the percentage of survivors (OD value with test compound divided by that of untreated control). Data analysis was completed using DataAspects Plate Manager analysis software (version 2001–2005, DataAspects Corporation, Los Gatos, CA, USA). Non-linear regression was used to obtain IC_50_ values.

### 4.7. Naegleria fowleri Culture and Cell Viability Assay

Activity against *Naegleria fowleri* was screened using a patient isolate from 1969 (ATCC 30215). Previously published methods using the AlamarBlue (Bio Rad, Hercules, CA, USA) colorimetric assay with axenically grown trophozoites were followed. A Biomek 3000 automated liquid-handling workstation (Beckman Coulter, Miami, FL, USA) was used to serially dilute the extracts to 50 and 5 µg/mL in Nelson’s culture media (Sigma-Aldrich, St. Louis, MO, USA) with a final concentration of 1% DMSO. The Biomek workstation was used to transfer diluted extracts, followed by the addition of 100,000 or 3000 *N. fowleri* trophozoites/well in 96- or 384-well screening plates, respectively [48].

### 4.8. Data Analysis

To facilitate the direct comparison of the results, extracts were given a simple “active/non-active” designation in each of the assays described above. The ESKAPE pathogens’ MICs were transformed into a scaled score for each pathogen by dividing and summing the highest-tested concentration (200 µg/mL) by each concentration in which activity was seen. (e.g., a sample active at 100 µg/mL would receive a scaled score of 3; (200/200) + (200/100) = 3.) For simplicity, activity was evaluated as a single, combined scaled score for all 6 pathogens. An extract was considered active if it had a scaled score ≥ 9. Only extracts exhibiting an IC_50_ value < 1 μg/mL in the infected macrophage model of the *L. donovani* assays were ranked as active. *M. tuberculosis* inhibition was noted as active when an extract inhibited ≥85% of bacterial growth, and activity against *N. fowleri* was defined as inhibition of >33% at any of two concentrations tested (50 and 5 μg/mL). Any cytotoxicity against the J774 macrophage cells (from the *L. donovani* infected macrophage assay) up to 20 μg/mL was categorized as active.

## Supplementary Materials

The following are available online at http://www.mdpi.com/1660-3397/16/10/376/s1, Table S1: MIC and cytotoxicity for ESKAPE pathogens treated with mangrove fungal extracts, Table S2: Growth inhibition and cytotoxicity of mangrove extracts inhibiting *M. tuberculosis*, Table S3: Inhibitory concentration and cytotoxicity of mangrove extracts toward *Leishmania donovani*, Table S4: Sensitivity (percent inhibition and cytotoxicity) of *Naegleria fowleri* to treatment with mangrove endophytic fungal extracts, Table S5: Screening data from 286 mangrove endophytic fungal extracts active in one or more pathogen screen, Table S6: Strains, treatment and bioactivity of mangrove endophytic fungi.
