# Supplementary material for: Exploitation of Mangrove Endophytic Fungi for Infectious Disease Drug Discovery
Source: Mar Drugs. 2018 Oct 10;16(10):376. doi: 10.3390/md16100376 (PMC6212984; doi:10.3390/md16100376)
Supplement: Supplementary file 1 [file marinedrugs-16-00376-s001.pdf]

Supplemental Information

Exploitation of mangrove endophytic fungi for infectious disease drug discovery

Danielle H. Demers[1], Matthew A. Knestrick[1], Renee Fleeman[2], Rahmy Tawfik[2], Ala Azhari[3], Ashley Souza[3], Brian Vesely[3], Mandy Netherton[4], Rashmi Gupta[4], Beatrice L. Colon[5], Christopher A. Rice[3], Mario A. Rodríguez-Pérez[6], Kyle H. Rohde[4], Dennis E. Kyle[3], Lindsey N. Shaw[2] and Bill J. Baker[1,*]

[1] Department of Chemistry and Center for Drug Discovery and Innovation, University of South Florida, Tampa, FL 33620, United States
[2] Department of Cell Biology, Microbiology and Molecular Biology, University of South Florida, Tampa, FL 33620, United States
[3] Department of Global Health, University of South Florida, Tampa, FL 33613, United States
[4] Division of Immunity and Pathogenesis, Burnett School of Biomedical Sciences, University of Central Florida, Orlando, FL 32827, United States
[5] Department of Molecular Medicine, University of South Florida, Tampa, FL 33613, United States
[6] Instituto Politécnico Nacional, Centro de Biotecnología Genómica, Blvd. del Maestro esq. Elías Piña s/n. 88710, Reynosa, Tamaulipas, Mexico.
* Correspondence: bjbaker@usf.edu

Contents

Table S1. MIC and cytotoxicity for ESKAPE pathogens treated with mangrove fungal extracts..2

Table S2. Growth inhibition and cytotoxicity of mangrove extracts inhibiting *M. tuberculosis*....4

Table S3. Inhibitory concentration and cytotoxicity of mangrove extracts toward *Leishmania donovani*........5

Table S4. Sensitivity (percent inhibition and cytotoxicity) of *Naegleria fowleri* to treatment with mangrove endophytic fungal extracts........7

Table S5. Screening data from 286 mangrove endophytic fungal extracts active in one or more pathogen screen........8

Table S6. Strains, treatment and bioactivity of mangrove endophytic fungi........13

Table S1. MIC and cytotoxicity for ESKAPE pathogens treated with mangrove fungal extracts.[a]

		Scaled		MIC (μg/mL) against Select Strains						Scaled Score Multiplier[d]					
**Sample ID[b]**	**Treatment[c]**	**Score[d] [46]**	**Cytotoxicity[e] IC_{50}**	**200**	**100**	**50**	**25**	**10**	**1**	**1**	**2**	**4**	**8**	**20**	**40**
TAP14-34A-4	DNMT	96	>50	ESKAPE	SKA	EA	EA	A	A	6	3	2	2	1	1
CQ10-30A-4	CONTROL	82	8.4	ES	ES	ES	S	S	S	2	2	2	1	1	1
CQ10-30A-4	DNMT	75	7.0	S	S	S	S	S	S	1	1	1	1	1	1
CQ10-30A-4	HDAC	75	7.2	S	S	S	S	S	S	1	1	1	1	1	1
HF14-11A-2	CONTROL	75	>50	S	S	S	S	S	S	1	1	1	1	1	1
HF14-11A-2	HDAC	75	>50	S	S	S	S	S	S	1	1	1	1	1	1
HF14-37B-1B	DNMT	75	>50	S	S	S	S	S	S	1	1	1	1	1	1
HF14-5A-1	CONTROL	75	>50	S	S	S	S	S	S	1	1	1	1	1	1
KML12-19MG-C4	CONTROL	75	9.1	S	S	S	S	S	S	1	1	1	1	1	1
KML12-19MG-C4	HDAC	75	11	S	S	S	S	S	S	1	1	1	1	1	1
KML12-19MG-C5	DNMT	75	20	S	S	S	S	S	S	1	1	1	1	1	1
TAP14-218C-2	CONTROL	75	42	S	S	S	S	S	S	1	1	1	1	1	1
TAP14-218C-2	DNMT	75	>50	S	S	S	S	S	S	1	1	1	1	1	1
TAP14-218C-2	HDAC	75	44	S	S	S	S	S	S	1	1	1	1	1	1
TAP14-34A-4	CONTROL	55	>50	ESKAP	EKA	EA	EA	A		5	3	2	2	1	0
HF14-39B-7	CONTROL	35	>50	S	S	S	S	S		1	1	1	1	1	0
HF14-5A-1	HDAC	35	>50	S	S	S	S	S		1	1	1	1	1	0
KML12-11MG-C5	HDAC	35	32	S	S	S	S	S		1	1	1	1	1	0
KML12-17MG-C1	CONTROL	35	>50	S	S	S	S	S		1	1	1	1	1	0
KML12-17MG-C1	DNMT	35	>50	S	S	S	S	S		1	1	1	1	1	0
KML12-19MG-C4	DNMT	35	>50	S	S	S	S	S		1	1	1	1	1	0
KML12-19MG-C5	HDAC	35	>50	S	S	S	S	S		1	1	1	1	1	0
KML12-2MG-C1	CONTROL	35	37	S	S	S	S	S		1	1	1	1	1	0
KML12-2MG-C1	DNMT	35	>50	S	S	S	S	S		1	1	1	1	1	0
KML12-2MG-C1	HDAC	35	>50	S	S	S	S	S		1	1	1	1	1	0
KML12-8MG-C2	CONTROL	35	30	S	S	S	S	S		1	1	1	1	1	0
KML12-8MG-C2	DNMT	35	>50	S	S	S	S	S		1	1	1	1	1	0
KML12-8MG-C2	HDAC	35	46	S	S	S	S	S		1	1	1	1	1	0
KML12-19MG-C1	DNMT	16	36	SA	S	S	S			2	1	1	1	0	0
TAP14-158B-2B II	DNMT	16	>50	ESAP	EA	EA				4	2	2	0	0	0
TAP14-34A-4	HDAC	16	>50	ESAP	EA	EA				4	2	2	0	0	0
EG12-40B-3	DNMT	15	>50	S	S	S	S			1	1	1	1	0	0
HF14-16C-2	DNMT	15	>50	S	S	S	S			1	1	1	1	0	0
HF14-16C-2	HDAC	15	>50	S	S	S	S			1	1	1	1	0	0
HF14-24D-2	HDAC	15	>50	S	S	S	S			1	1	1	1	0	0
HF14-37B-1B	HDAC	15	>50	S	S	S	S			1	1	1	1	0	0
HF14-39B-7	DNMT	15	>50	S	S	S	S			1	1	1	1	0	0
HF14-7C-5A	HDAC	15	>50	S	S	S	S			1	1	1	1	0	0
HF14-9A-1	DNMT	15	>50	S	S	S	S			1	1	1	1	0	0

Sample ID[b]	Treatment[c]	Scaled Score[d] [46]	Cytotoxicity[e] IC_{50}	MIC (μg/mL) against Select Strains						Scaled Score Multiplier[d]					
				200	100	50	25	10	1	1	2	4	8	20	40
KML12-11MG-C1	DNMT	15	40	S	S	S	S			1	1	1	1	0	0
KML12-11MG-C5	DNMT	15	33	S	S	S	S			1	1	1	1	0	0
KML12-17MG-C1	HDAC	15	>50	S	S	S	S			1	1	1	1	0	0
KML12-19MG-C1	HDAC	15	29	S	S	S	S			1	1	1	1	0	0
KML12-4MG-C4	HDAC	15	37	S	S	S	S			1	1	1	1	0	0
KML12-8MG-C5	HDAC	15	>50	S	S	S	S			1	1	1	1	0	0
KML12-14MG-B2a	CONTROL	9	>50	EAP	EAP					3	3	0	0	0	0

[a]Blank cells indicate no inhibition. Ordered by decreasing scaled score. [b]Strain name includes location of mangrove source (CQ, Coquina Beach, Sarasota, FL; EG, Everglades City, FL; HF, Howard Franklin Causeway, Tampa, FL; KML, Keys Marine Lab, Layton, FL; TAP, Tapachula, MX), year, isolation plate sequence. [c]Control, no epigenetic regulation treatment; DNMT, treated with DNA methyltransferase inhibitor 5-azacytidine; HDAC, treated with histone deacetylase inhibitor sodium butyrate. [d]Scaled Score [46] calculated by dividing and summing the highest tested concentration (200 μg/mL) by each concentration in which activity was seen. For example a sample active at 100 μg/mL would receive a scaled score of 3; (200/200) + (100/200) = 3. For convenience, the Scaled Score multiplier takes the place of the fractions. [e]J774 cell line.

Table S2. Growth inhibition and cytotoxicity of mangrove extracts inhibiting *M. tuberculosis.*[a]

Sample ID[b]	Treatment[c]	GI (%)	Cytotoxicity[d]	Sample ID[b]	Treatment[c]	GI (%)	Cytotoxicity[d]
TAP14-218C-2	DNMT	107.3	50	HF14-38D-1	HDAC	100.1	>50
TAP14-198D-7	DNMT	107.1	>50	EG12-25B-7B	HDAC	100.1	>50
TAP14-218C-2	HDAC	107.1	44	TAP14-202C-4	HDAC	100.1	>50
TAP14-145C-1	DNMT	106.8	>50	TAP14-183C-2	CONTROL	100.1	>50
TAP14-218C-2	CONTROL	106.5	42	HF14-31A-1	DNMT	99.9	>50
TAP14-147B-4	HDAC	105.7	>50	HF14-39B-6	DNMT	99.6	>50
TAP14-147B-4	DNMT	105.2	>50	HF14-40D-4	CONTROL	99.5	>50
EG10-15A-2	CONTROL	105.1	10	HF14-52C-8	HDAC	99.4	>50
TAP14-198D-7	HDAC	105.0	>50	HF14-52C-8	DNMT	99.3	>50
HF14-6A-3	DNMT	104.2	>50	TAP14-132B-2	HDAC	99.2	18
TAP14-34A-4	DNMT	104.2	>50	HF14-24B-3	CONTROL	99.1	>50
TAP14-114A-2	CONTROL	104.1	>50	HF14-38D-2	HDAC	99.0	>50
TAP14-114A-2	HDAC	104.0	>50	KML12-2MG-F4	DNMT	98.9	>50
TAP14-228D-2	HDAC	103.9	>50	HF14-27D-2	HDAC	98.9	>50
TAP14-114A-2	DNMT	103.8	>50	HF14-32B-4	HDAC	98.9	>50
TAP14-198D-5A II	DNMT	103.8	>50	HF14-23D-2	CONTROL	98.8	>50
TAP14-202C-3	CONTROL	103.7	>50	HF14-1D-3	CONTROL	98.4	>50
TAP14-190D-3A	HDAC	103.7	>50	TAP14-183C-2	DNMT	98.3	>50
TAP14-202C-1	HDAC	103.1	>50	HF14-24B-3	DNMT	98.3	>50
TAP14-190D-3A	CONTROL	103.0	>50	TAP14-226B-B	CONTROL	97.9	>50
HF14-24D-2	DNMT	102.9	>50	EG12-30B-3B	HDAC	97.9	5.7
EG10-15A-2	DNMT	102.8	14	HF14-19C-1	DNMT	97.8	>50
TAP14-147B-4	CONTROL	102.8	>50	TAP14-220D-1	CONTROL	97.7	>50
HF14-14B-2	CONTROL	102.7	>50	TAP14-210B-1	DNMT	97.7	>50
TAP14-215B1-1	HDAC	102.5	>50	CC13-109A-13B	CONTROL	97.7	>50
HF14-8D-1	HDAC	102.3	>50	HF14-22C-9	CONTROL	97.5	>50
TAP14-111A-4B	DNMT	102.2	8.6	HF14-14A-1	HDAC	97.3	>50
EG10-22C-1	HDAC	102.2	>50	EG12-28D-2	CONTROL	97.3	26
TAP14-111A-4B	HDAC	102.1	>50	EG12-20E-3B	HDAC	97.2	24
TAP14-147C-5A	DNMT	101.9	>50	TAP14-190D-3A	DNMT	97.1	>50
TAP14-216C-5	CONTROL	101.9	>50	EG12-28D-2	HDAC	97.0	26
HF14-35B-5B	HDAC	101.7	>50	HF14-22C-9	HDAC	97.0	>50
EG10-15A-2	HDAC	101.7	10	EG12-30B-3B	CONTROL	96.9	13
EG12-40B-3	DNMT	101.7	>50	HF14-1D-3	HDAC	96.9	>50
EG10-22C-1	CONTROL	101.6	8.9	EG12-29A-3	DNMT	96.9	>50
TAP14-215B1-1	CONTROL	101.6	33	HF14-1D-2	CONTROL	96.8	>50
EG10-22C-1	DNMT	101.6	32	HF14-39B-6	HDAC	96.8	>50
CQ10-28B-3	DNMT	101.5	8.4	HF14-14B-3	CONTROL	96.7	>50
CQ10-28B-3	HDAC	101.2	5.8	EG12-28D-2	DNMT	96.7	>50
CQ10-30A-4	HDAC	101.2	7.2	CQ10-27C-4B	DNMT	96.5	25
HF14-15C-4B	HDAC	100.9	>50	TAP14-198D-5A II	CONTROL	96.4	>50
EG12-27D-4	CONTROL	100.9	>50	TAP14-226B-B	HDAC	96.3	>50
EG12-24A-2A	CONTROL	100.8	16	HF14-30C-2C	HDAC	96.2	>50
TAP14-202C-4	DNMT	100.7	>50	TAP14-216B-1	HDAC	96.0	>50
CQ10-30A-4	DNMT	100.6	7.0	TAP14-211B-1	DNMT	95.8	>50
CQ10-30A-4	CONTROL	100.6	8.4	KML12-26MG-E1	CONTROL	95.6	>50
TAP14-212D-2	CONTROL	100.5	>50	EG12-22D-1	CONTROL	95.4	26
HF14-28B-2	DNMT	100.4	>50	TAP14-203C-21	DNMT	95.3	28
TAP14-178D-1A	DNMT	100.3	>50	EG12-29A-3	HDAC	95.1	>50
HF14-28B-2	HDAC	100.3	>50	EG12-31C-1	CONTROL	95.0	>50

[a]Ordered by decreasing GI. [b]Strain name includes location of mangrove source (CC, Courtney Campbell Causeway, Hillsborough, FL; CQ, Coquina Beach, Sarasota, FL; EG, Everglades City, FL; HF, Howard Franklin Causeway, Tampa, FL; KML, Keys Marine Lab, Layton, FL; TAP, Tapachula, MX), year, isolation plate sequence. [c]Control, no epigenetic regulation treatment; DNMT, treated with DNA methyltransferase inhibitor 5-azacytidine; HDAC, treated with histone deacetylase inhibitor sodium butyrate.

Table S3. Inhibitory concentration and cytotoxicity of mangrove extracts toward *Leishmania donovani*.[a]

Sample[b]	Treatment[c]	IC_{50}[d]	Cytotoxicity[e]	Sample[b]	Treatment[c]	IC_{50}[d]	Cytotoxicity[e]
TAP14-215B1-1	HDAC	0.09	50	TAP14-165D-1A	CONTROL	0.61	>50
TAP14-211B-1	DNMT	0.11	>50	TAP14-160D-4	CONTROL	0.61	>50
EG12-25B-7A	DNMT	0.24	25	EG10-22C-1	CONTROL	0.61	8.9
TAP14-210D-1	HDAC	0.26	>50	HF14-30D-2	DNMT	0.61	>50
BGC13-CR-1B	HDAC	0.26	>50	KML12-19MG-C1	CONTROL	0.61	28
TAP14-208D-1B	HDAC	0.27	>50	HF14-22C-2B	CONTROL	0.61	>50
TAP14-166B-1B	CONTROL	0.27	>50	TAP14-145C-2	HDAC	0.62	>50
TAP14-216C-5	HDAC	0.27	>50	HF14-1D-2	HDAC	0.62	>50
TAP14-206C-5B	DNMT	0.27	>50	KML12-19MG-C1	DNMT	0.62	36
TAP14-212D-2	CONTROL	0.28	>50	HM13-14B-3	CONTROL	0.62	>50
TAP14-147D-5B	CONTROL	0.28	>50	EG12-3C-1	DNMT	0.62	>50
BGC11-58B-5	HDAC	0.28	>50	HF14-35B-5C	CONTROL	0.62	>50
TAP14-214D-1B	DNMT	0.28	>50	TAP14-147B-4	CONTROL	0.62	>50
HF14-21D-2B	DNMT	0.28	>50	EG12-30B-3B	CONTROL	0.63	13
TAP14-190D-3B	HDAC	0.29	>50	EG10-21D-1	CONTROL	0.63	>50
TAP14-206C-5B	HDAC	0.29	>50	TAP14-216B-1	DNMT	0.63	>50
TAP14-230C1-1	CONTROL	0.29	>50	TAP14-227B-1A	CONTROL	0.64	>50
TAP14-198D-7	DNMT	0.29	>50	HF14-5A-1	CONTROL	0.64	>50
EG10-22A-3	CONTROL	0.29	>50	HF14-24C-9A	DNMT	0.64	>50
TAP14-202C-4	HDAC	0.29	>50	HF14-34B-1B	HDAC	0.64	>50
TAP14-165D-1A	DNMT	0.29	28	TAP14-132B-2	DNMT	0.64	30
TAP14-147D-5B	HDAC	0.29	>50	TAP14-183C-5	CONTROL	0.64	>50
TAP14-212D-2	DNMT	0.29	>50	BGC11-54B-5	DNMT	0.65	>50
TAP14-211D-1	HDAC	0.29	>50	TAP14-156D-1B	DNMT	0.65	>50
EG12-3C-2 I	DNMT	0.30	>50	HF14-6A-3	DNMT	0.65	>50
TAP14-226B-A	DNMT	0.30	>50	HF14-24C-9A	CONTROL	0.65	>50
TAP14-220D-1	DNMT	0.30	>50	EG12-25B-7A	HDAC	0.66	>50
TAP14-218C-2	HDAC	0.30	44	HF14-5A-1	HDAC	0.66	>50
TAP14-180D-3A	CONTROL	0.30	>50	EG12-30B-3B	DNMT	0.68	14
TAP14-213C-1	HDAC	0.30	>50	HF14-23C-3B	DNMT	0.68	>50
TAP14-211B-1	CONTROL	0.30	>50	HF14-52C-8	CONTROL	0.69	>50
EG12-3C-2 I	HDAC	0.30	>50	EG12-30B-3B	HDAC	0.75	5.7
HF14-21D-2B	HDAC	0.30	>50	CC13-7CR-B11A	DNMT	0.78	>50
CC13-1P-F1	CONTROL	0.30	>50	HF14-35C-3A	HDAC	0.80	>50
EG12-12D-3	CONTROL	0.31	>50	EG10-18A-2	CONTROL	0.83	>50
CC13-1P-F1	DNMT	0.31	>50	HF14-25C-4B	DNMT	0.84	>50
TAP14-16C-3	DNMT	0.31	>50	TAP14-203C-21	DNMT	0.87	28
CC13-7CR-T12	CONTROL	0.31	>50	TAP14-180D-3A	DNMT	0.87[d]	>50
TAP14-142C-4B	CONTROL	0.31	>50	TAP14-198D-7	CONTROL	0.88	>50
TAP14-147B-4	HDAC	0.31	>50	KML12-19MG-C5	DNMT	0.88	20
EG10-15D-2	CONTRO	0.31	>50	TAP14-203C-21	HDAC	0.88	25
TAP14-216C-5	DNMT	0.31	>50	HF14-27C-7	HDAC	0.88	>50
TAP14-212D-2	HDAC	0.31	>50	HF14-28B-2	DNMT	0.88	>50
EG12-10E-1	CONTROL	0.31	>50	TAP14-157B-2	HDAC	0.88	>50
TAP14-215B1-1	CONTROL	0.35	33	TAP14-202C-1	HDAC	0.88	>50
EG12-12D-3	HDAC	0.40	>50	HF14-27C-7	CONTROL	0.88	>50
TAP14-145C-2	DNMT	0.41	>50	CC13-7CR-T12	HDAC	0.88	>50
TAP14-227B-1A	DNMT	0.48	>50	TAP14-215B1-1	DNMT	0.89	>50
TAP14-208D-1B	CONTROL	0.50	>50	HF14-35B-5C	HDAC	0.89	>50
TAP14-197C-5	DNMT	0.56	>50	EG12-25B-7A	CONTROL	0.89	>50
TAP14-196B-5	HDAC	0.57	>50	TAP14-226B-A	CONTROL	0.89	26
TAP14-206C-1A	HDAC	0.59	>50	CQ10-33C-3	DNMT	0.89	>50
HF14-6A-3	CONTROL	0.59	>50	KML12-18MG-C2	CONTROL	0.90	>50
CQ10-29C-3	CONTROL	0.60	>50	EG12-3C-2 II	DNMT	0.90	>50
EG12-20E-3B	HDAC	0.60	24	HF14-8D-3	CONTROL	0.93	>50
TAP14-3D-2D	CONTROL	0.60	>50	TAP14-224B-1	DNMT	0.94	>50

HF14-30A-4A	DNMT	0.61	>50	HF14-30D-2	HDAC	0.96	>50
TAP14-210D-1	CONTROL	0.61	>50	EG12-28D-2	DNMT	0.98	>50

[a]Ordered by decreasing IC_{50}. [b]Strain name includes location of mangrove source (CC, Courtney Campbell Causeway, Hillsborough, FL; CQ, Coquina Beach, Sarasota, FL; EG, Everglades City, FL; HF, Howard Franklin Causeway, Tampa, FL; KML, Keys Marine Lab, Layton, FL; TAP, Tapachula, MX), year, isolation plate sequence. [c]Control, no epigenetic regulation treatment; DNMT, treated with DNA methyltransferase inhibitor 5-azacytidine; HDAC, treated with histone deacetylase inhibitor sodium butyrate. [d]µg/mL. [e]J774 macrophage cell line.

Table S4. Sensitivity (percent inhibition and cytotoxicity) of *Naegleria fowleri* to treatment with mangrove endophytic fungal extracts.[a]

		Inhibition		
**Sample[b]**	**Treatment[c]**	**%**	**µg/mL**	**Cytotoxicity[d]**
CQ10-27B-1	HDAC	>33	5	20
CQ10-30A-4	CONTROL	>33	27.5	8.4
CQ10-30A-4	DNMT	>33	27.5	7.0
CQ10-30A-4	HDAC	>33	27.5	7.2
HF14-2D-1	CONTROL	>67	50	
HF14-43C-2B	CONTROL	>67	50	
CQ10-28B-3	DNMT	>33	50	8.4
CQ10-29C-4	CONTROL	>33	50	
EG12-24A-2A	CONTROL	>33	50	16
EG12-25B-5	CONTROL	>33	50	
EG12-27C-3	HDAC	>33	50	50
EG12-28C-1	CONTROL	>33	50	21
EG12-28D-2	CONTROL	>33	50	26
EG12-31A-3B	HDAC	>33	50	50
EG12-34B-6	CONTROL	>33	50	
EG12-37B-1	CONTROL	>33	50	
EG12-8A-3	CONTROL	>33	50	11
EG12-8A-3	DNMT	>33	50	50
EG12-8A-3	HDAC	>33	50	13
HF14-16C-2A	CONTROL	>33	50	50
HF14-23D-3	HDAC	>33	50	
HF14-24C-9A	DNMT	>33	50	50
HF14-37B-1B	CONTROL	>33	50	50
HF14-37B-1B	DNMT	>33	50	50
HF14-37B-1B	HDAC	>33	50	50
HF14-42C-5B	CONTROL	>33	50	50
HF14-42C-5B	DNMT	>33	50	50
HF14-42C-5B	HDAC	>33	50	50
HF14-51C-4	CONTROL	>33	50	
HF14-7C-1	CONTROL	>33	50	
HF14-7C-5B	CONTROL	>33	50	
HM13-23B-2D	CONTROL	>33	50	

[a]Ordered by concentration. [b]Strain name includes location of mangrove source (CQ, Coquina Beach, Sarasota, FL; EG, Everglades City, FL; HF, Howard Franklin Causeway, Tampa, FL), year, isolation plate sequence. [c]Control, no epigenetic regulation treatment; DNMT, treated with DNA methyltransferase inhibitor 5-azacytidine; HDAC, treated with histone deacetylase inhibitor sodium butyrate. [d]J774 macrophage cell line.

Table S5. Screening data from 286 mangrove endophytic fungal extracts active in one or more pathogen screen.[a]

Sample Name[b]	Treatment[c]	ESKAPE Scaled Score	*M. tuberculosis* % Inhibition	*L. donovani* IM IC_{50}[d] (μm)	*N. fowleri* % Inhibition	J774 IC_{50} (μm)	N'mber of Hits
BGC11-54B-5	DNMT			0.6		50	1
BGC11-58B-5	HDAC			0.3		50	1
BGC13-CR-1B	HDAC			0.3		50	1
CC13-109A-13B	CONTROL		97.7				1
CC13-1P-F1	CONTROL			0.3		50	1
CC13-1P-F1	DNMT			0.3		50	1
CC13-7CR-B11A	DNMT			0.8		50	1
CC13-7CR-T12	CONTROL			0.3		50	1
CC13-7CR-T12	HDAC			0.9		50	1
CQ10-27B-1	HDAC				33	20	1
CQ10-27C-4B	DNMT		96.5			25	1
CQ10-28A-3	HDAC			1.2		16	1
CQ10-28B-3	CONTROL		101.6				1
CQ10-28B-3	DNMT		101.5		33		2
CQ10-28B-3	HDAC		101.2				1
CQ10-29C-3	CONTROL			0.6			1
CQ10-30A-4	CONTROL	82	100.6	1.3	33		4
CQ10-30A-4	DNMT	75	100.6		33		3
CQ10-30A-4	HDAC	75	101.2		33		3
CQ10-33C-3	DNMT			0.9		50	1
EG10-15A-2	DNMT		102.8	4.8		14	2
EG10-15A-2	HDAC		101.7			10	1
EG10-15A-2	CONTROL		105.1			10	1
EG10-15D-2	CONTROL			0.3		50	1
EG10-18A-2	CONTROL			0.8		50	1
EG10-21D-1	CONTROL			0.6		50	1
EG10-22A-3	CONTROL			0.3		50	1
EG10-22C-1	CONTROL		101.6	0.6			2
EG10-22C-1	DNMT		101.6	1.2		32	2
EG10-22C-1	HDAC		102.2				1
EG10-37C-4	CONTROL		98.2	0.6			2
EG10-37C-4	HDAC		98.1	1.0			2
EG12-10E-1	CONTROL			0.3		50	1
EG12-10E-1	DNMT			4.9		50	1
EG12-12D-3	CONTROL			0.3		50	1
EG12-12D-3	HDAC			0.4		50	1
EG12-20E-3B	HDAC		97.2	0.6		24	2
EG12-22D-1	CONTROL		95.4			26	1
EG12-24A-2A	CONTROL		100.8	3.5	33	16	3
EG12-25B-5	CONTROL				33		1
EG12-25B-7A	DNMT			0.2		25	1
EG12-25B-7A	HDAC			0.7		50	1
EG12-25B-7A	CONTROL			0.9		50	1
EG12-25B-7B	HDAC		100.1				1
EG12-27C-3	CONTROL				33		1
EG12-27C-3	HDAC				33	50	1
EG12-27D-4	CONTROL		100.9				1
EG12-28C-1	CONTROL			1.8	33	21	2
EG12-28D-2	CONTROL		97.3		33	26	2
EG12-28D-2	HDAC		97.0	3.3		26	2
EG12-28D-2	DNMT		96.7	1.0		50	2
EG12-29A-3	DNMT		96.9			50	1
EG12-29A-3	HDAC		95.1			50	1
EG12-30B-3B	CONTROL		96.9	0.6		13	2

Sample Name[b]	Treatment[c]	ESKAPE Scaled Score	*M. tuberculosis* % Inhibition	*L. donovani* IM IC_{50}[d] (μm)	*N. fowleri* % Inhibition	J774 IC_{50} (μm)	N'mber of Hits
EG12-30B-3B	HDAC		97.9	0.8			2
EG12-30B-3B	DNMT			0.7		14	1
EG12-31A-3B	HDAC				33	50	1
EG12-31C-1	CONTROL		95.0				1
EG12-34B-6	CONTROL				33		1
EG12-37B-1	CONTROL				33		1
EG12-3C-1	DNMT			0.6		50	1
EG12-3C-2 I	DNMT			0.3		50	1
EG12-3C-2 I	HDAC			0.3		50	1
EG12-3C-2 II	DNMT			0.9		50	1
EG12-40B-3	CONTROL		97.3	2.6	33		3
EG12-40B-3	DNMT	15	101.7	1.3		50	3
EG12-40B-3	HDAC	15	101.4	1.3			3
EG12-8A-3	CONTROL				33	11	1
EG12-8A-3	DNMT				33	50	1
EG12-8A-3	HDAC				33	13	1
HF14-11A-2	CONTROL	75		4.8		50	2
HF14-11A-2	DNMT			4.0		50	1
HF14-11A-2	HDAC	75		4.6		50	2
HF14-14A-1	HDAC		97.3				1
HF14-14B-2	CONTROL		102.7			50	1
HF14-14B-3	CONTROL		96.7				1
HF14-15C-4B	HDAC		100.9				1
HF14-16C-2	CONTROL			2.0		50	1
HF14-16C-2	DNMT	15		3.5		50	2
HF14-16C-2	HDAC	15		2.5		50	2
HF14-16C-2A	DNMT			4.9		50	1
HF14-16C-2AL	CONTROL			2.7	33	50	2
HF14-19C-1	DNMT		97.8				1
HF14-1D-2	CONTROL		96.8	3.1		50	2
HF14-1D-2	HDAC			0.6		50	1
HF14-1D-3	CONTROL		98.4	3.2		50	2
HF14-1D-3	HDAC		96.9			50	1
HF14-21D-2B	DNMT			0.3		50	1
HF14-21D-2B	HDAC			0.3		50	1
HF14-22C-2B	CONTROL			0.6		50	1
HF14-22C-9	CONTROL		97.5				1
HF14-22C-9	HDAC		97.0				1
HF14-23C-3B	DNMT			0.7		50	1
HF14-23D-2	CONTROL		98.8				1
HF14-23D-3	HDAC				33		1
HF14-24B-3	DNMT		98.3				1
HF14-24B-3	CONTROL		99.1				1
HF14-24C-9A	CONTROL			0.7		50	1
HF14-24C-9A	DNMT			0.6	33	50	2
HF14-24D-2	DNMT		102.9				1
HF14-24D-2	HDAC	15					1
HF14-25C-4B	DNMT			0.8		50	1
HF14-27C-7	CONTROL			0.9		50	1
HF14-27C-7	HDAC			0.9		50	1
HF14-27D-2	HDAC		98.9				1
HF14-28B-2	DNMT		100.4	0.9		50	2
HF14-28B-2	HDAC		100.3			50	1
HF14-2D-1	CONTROL				33		1
HF14-30A-4A	DNMT			0.6		50	1
HF14-30C-2C	HDAC		96.2				1

Sample Name[b]	Treatment[c]	ESKAPE Scaled Score	*M. tuberculosis* % Inhibition	*L. donovani* IM IC_{50}[d] (µm)	*N. fowleri* % Inhibition	J774 IC_{50} (µm)	N'mber of Hits
HF14-30D-2	DNMT			0.6		50	1
HF14-30D-2	HDAC			1.0		50	1
HF14-30D-5	CONTROL			1.3		50	1
HF14-31A-1	DNMT		99.9				1
HF14-32B-4	HDAC		98.9				1
HF14-34B-1B	HDAC			0.6		50	1
HF14-35B-5B	HDAC		101.7	3.6		50	2
HF14-35B-5C	CONTROL			0.6		50	1
HF14-35B-5C	HDAC			0.9		50	1
HF14-35C-3A	HDAC			0.8		50	1
HF14-37B-1B	CONTROL			3.0	33	50	2
HF14-37B-1B	HDAC	15		3.1	33	50	3
HF14-37B-1B	DNMT	75		1.3	33	50	3
HF14-38D-1	HDAC		100.1	1.3		50	2
HF14-38D-2	HDAC		99.0				1
HF14-39B-6	HDAC		96.8				1
HF14-39B-6	DNMT		99.6				1
HF14-39B-7	CONTROL	35		3.3		50	2
HF14-39B-7	DNMT	15				50	1
HF14-40D-4	CONTROL		99.5				1
HF14-42C-5B	CONTROL			3.8	33	50	2
HF14-42C-5B	HDAC			1.8	33	50	2
HF14-42C-5B	DNMT				33	50	1
HF14-43C-2B	CONTROL				33		1
HF14-51C-4	CONTROL				33		1
HF14-52C-8	CONTROL			0.7		50	1
HF14-52C-8	HDAC		99.4	1.8		50	2
HF14-52C-8	DNMT		99.3	2.5		50	2
HF14-5A-1	CONTROL	75		0.6		50	2
HF14-5A-1	HDAC	35		0.7		50	2
HF14-5A-1	DNMT			3.5		50	1
HF14-6A-3	CONTROL			0.6		50	1
HF14-6A-3	DNMT		104.2	0.7		50	2
HF14-7C-1	CONTROL				33		1
HF14-7C-5A	HDAC	15				50	1
HF14-7C-5B	CONTROL				33		1
HF14-8D-1	HDAC		102.3				1
HF14-8D-3	CONTROL			0.9		50	1
HF14-9A-1	DNMT	15				50	1
HM13-14B-3	CONTROL			0.6		50	1
HM13-23b-2d	CONTROL				33		1
KML12-11MG-C1	DNMT	15				40	1
KML12-11MG-C5	HDAC	35				32	1
KML12-11MG-C5	DNMT	15					1
KML12-17MG-C1	CONTROL	35				50	1
KML12-17MG-C1	HDAC	15				50	1
KML12-17MG-C1	DNMT	35				50	1
KML12-18MG-C2	CONTROL			0.9		50	1
KML12-19MG-C1	CONTROL			0.6		28	1
KML12-19MG-C1	HDAC	15		1.2		29	2
KML12-19MG-C1	DNMT	16		0.6		36	2
KML12-19MG-C4	CONTROL	75		1.8			2
KML12-19MG-C4	HDAC	75		3.5		11	2
KML12-19MG-C4	DNMT	35		4.9		50	2
KML12-19MG-C5	HDAC	35		1.2		50	2
KML12-19MG-C5	DNMT	75		0.9		20	2

**Sample Name[b]**	**Treatment[c]**	**ESKAPE Scaled Score**	***M. tuberculosis* % Inhibition**	***L. donovani* IM IC_{50}[d] (µm)**	***N. fowleri* % Inhibition**	**J774 IC_{50} (µm)**	**N'mber of Hits**
KML12-26MG-E1	CONTROL		95.6				1
KML12-2MG-C1	CONTROL	35					1
KML12-2MG-C1	HDAC	35				50	1
KML12-2MG-C1	DNMT	35				50	1
KML12-2MG-F4	DNMT		98.9				1
KML12-4MG-C4	HDAC	15		4.9		37	2
KML12-6MG-C3	HDAC			2.6		19	1
KML12-8MG-C2	CONTROL	35		3.6		30	2
KML12-8MG-C2	HDAC	35		2.6		46	2
KML12-8MG-C2	DNMT	35		1.6		50	2
KML12-8MG-C5	HDAC	15				50	1
KML12-8MG-C5	DNMT			3.6		50	1
TAP14-111A-4B	HDAC		102.1	2.7		50	2
TAP14-111A-4B	DNMT		102.2	1.8		50	2
TAP14-114A-2	CONTROL		104.1				1
TAP14-114A-2	HDAC		104.0				1
TAP14-114A-2	DNMT		103.8				1
TAP14-132B-2	HDAC		99.2			18	1
TAP14-132B-2	DNMT			0.6		30	1
TAP14-142C-4B	CONTROL			0.3		50	1
TAP14-145C-1	DNMT		106.8	1.2		50	2
TAP14-145C-2	HDAC			0.6		50	1
TAP14-145C-2	DNMT			0.4		50	1
TAP14-147B-4	CONTROL		102.8	0.6		50	2
TAP14-147B-4	HDAC		105.7	0.3		50	2
TAP14-147B-4	DNMT		105.2	5.0		50	2
TAP14-147C-5A	DNMT		101.9				1
TAP14-147D-5B	CONTROL			0.3		50	1
TAP14-147D-5B	HDAC			0.3		50	1
TAP14-156D-1B	DNMT			0.6		50	1
TAP14-157B-2	HDAC			0.9		50	1
TAP14-158B-2B II	DNMT	16					1
TAP14-160D-4	CONTROL			0.6		50	1
TAP14-165D-1A	CONTROL			0.6		50	1
TAP14-165D-1A	DNMT			0.3		28	1
TAP14-166B-1B	CONTROL			0.3		50	1
TAP14-16C-3	DNMT			0.3		50	1
TAP14-178D-1A	DNMT		100.3				1
TAP14-180D-3A	CONTROL			0.3		50	1
TAP14-180D-3A	DNMT			0.9		50	1
TAP14-183C-2	CONTROL		100.1				1
TAP14-183C-2	DNMT		98.3				1
TAP14-183C-5	CONTROL			0.6		50	1
TAP14-190D-3A	CONTROL		103.0				1
TAP14-190D-3A	HDAC		103.7				1
TAP14-190D-3A	DNMT		97.1				1
TAP14-190D-3B	HDAC			0.3		50	1
TAP14-196B-5	HDAC			0.6		50	1
TAP14-197C-5	DNMT			0.6		50	1
TAP14-198D-5A II	CONTROL		96.4				1
TAP14-198D-5A II	DNMT		103.8				1
TAP14-198D-7	CONTROL			0.9		50	1
TAP14-198D-7	HDAC		105.0			50	1
TAP14-198D-7	DNMT		107.1	0.3		50	2
TAP14-202C-1	HDAC		103.1	0.9		50	2
TAP14-202C-3	CONTROL		103.7				1

Sample Name[b]	Treatment[c]	ESKAPE Scaled Score	*M. tuberculosis* % Inhibition	*L. donovani* IM IC_{50}[d] (μm)	*N. fowleri* % Inhibition	J774 IC_{50} (μm)	N'mber of Hits
TAP14-202C-4	HDAC		100.1	0.3		50	2
TAP14-202C-4	DNMT		100.7	2.7		50	2
TAP14-203C-21	HDAC			0.9		25	1
TAP14-203C-21	DNMT		95.3	0.9		28	2
TAP14-206C-1A	HDAC			0.6		50	1
TAP14-206C-5B	HDAC			0.3		50	1
TAP14-206C-5B	DNMT			0.3		50	1
TAP14-208D-1B	CONTROL			0.5		50	1
TAP14-208D-1B	HDAC			0.3		50	1
TAP14-210B-1	DNMT		97.7			50	1
TAP14-210D-1	CONTROL			0.6		50	1
TAP14-210D-1	HDAC			0.3		50	1
TAP14-211B-1	CONTROL			0.3		50	1
TAP14-211B-1	DNMT		95.8	0.1		50	2
TAP14-211D-1	HDAC			0.3		50	1
TAP14-212D-2	CONTROL		100.5	0.3		50	2
TAP14-212D-2	HDAC			0.3		50	1
TAP14-212D-2	DNMT			0.3		50	1
TAP14-213C-1	HDAC			0.3		50	1
TAP14-214D-1B	DNMT			0.3		50	1
TAP14-215B1-1	CONTROL		101.6	0.3		33	2
TAP14-215B1-1	HDAC		102.5	0.1		50	2
TAP14-215B1-1	DNMT			0.9		50	1
TAP14-216B-1	HDAC		96.0	2.4		50	2
TAP14-216B-1	DNMT			0.6		50	1
TAP14-216C-5	CONTROL		101.9	3.6		50	2
TAP14-216C-5	HDAC			0.3		50	1
TAP14-216C-5	DNMT			0.3		50	1
TAP14-218C-2	CONTROL	75	106.5	1.7		42	3
TAP14-218C-2	HDAC	75	107.1	0.3		44	3
TAP14-218C-2	DNMT	75	107.3			50	2
TAP14-220D-1	CONTROL		97.7	2.1		50	2
TAP14-220D-1	DNMT			0.3		50	1
TAP14-224B-1	DNMT			0.9		50	1
TAP14-226B-A	CONTROL			0.9		26	1
TAP14-226B-A	DNMT			0.3		50	1
TAP14-226B-B	CONTROL		97.9				1
TAP14-226B-B	HDAC		96.3				1
TAP14-227B-1A	CONTROL			0.6		50	1
TAP14-227B-1A	DNMT			0.5		50	1
TAP14-228D-2	HDAC		103.9				1
TAP14-230C1-1	CONTROL			0.3		50	1
TAP14-34A-4	CONTROL	55					1
TAP14-34A-4	HDAC	16					1
TAP14-34A-4	DNMT	96	104.2				2
TAP14-3D-2D	CONTROL			0.6		50	1

[a]Blank cells indicate inactive using assay-specific criteria (see manuscript). Alphabetical by strain name. [b]Strain name includes location of fungal source (BGC, Gulf of Mexico, FL; CC, Courtney Campbell Causeway, Tampa, FL; CQ, Coquina Beach, Sarasota, FL; EG, Everglades City, FL; HF, Howard Franklin Causeway, Tampa, FL; HM, Honeymoon Island, FL; KML, Keys Marine Lab, Layton, FL; TAP, Tapachula, MX), year, isolation plate sequence. [c]Control, no epigenetic regulation treatment; DNMT, treated with DNA methyltransferase inhibitor 5-azacytidine; HDAC, treated with histone deacetylase inhibitor sodium butyrate. [d]Scaled score, see Experimental Section. [e]IM IC_{50}, Infected Macrophage (J774) model of *L. donovani* assay.

Table S6. Strains, treatment and bioactivity of mangrove endophytic fungi.[a]

	Treatment,[c] Activity[d]				Treatment,[c] Activity[d]		
**Sample[b]**	**Control**	**DNMT**	**HDAC**	**Sample[b]**	**Control**	**DNMT**	**HDAC**
HF14-11A-2	E		E	TAP14-198D-7	L	LT	T
HF14-16C-2		E	E	TAP14-216B-1		L	T
HF14-24D-2		T	E	TAP14-226B-B	T		T
HF14-7C-5A			E	TAP14-228D-2			T
KML12-11MG-C5		E	E	CC13-109A-13B	T		
KML12-17MG-C1	E	E	E	CC13-1P-F1	L	L	
KML12-19MG-C1	L	EL	E	CC13-7CR-B11A		L	
KML12-19MG-C4	E	E	E	CQ10-27C-4B		T	
KML12-19MG-C5		LE	E	CQ10-29C-3	L		
KML12-2MG-C1	E	E	E	CQ10-29C-4	N		
KML12-4MG-C4			E	CQ10-33C-3		L	
KML12-8MG-C2	E	E	E	EG10-15D-2	L		
KML12-8MG-C5			E	EG10-18A-2	L		
TAP14-34A-4	E	ET	E	EG10-21D-1	L		
HF14-5A-1	EL		EL	EG10-22A-3	L		
TAP14-218C-2	ET	ET	ELT	EG12-10E-1	L		
HF14-37B-1B	N	EN	EN	EG12-22D-1	T		
BGC11-54B-5		L	L	EG12-24A-2A	NT		
BGC13-CR-1B			L	EG12-25B-5	N		
CC13-7CR-T12	L		L	EG12-27D-4	T		
EG12-12D-3	L		L	EG12-28C-1	N		
EG12-25B-7A	L	L	L	EG12-31C-1	T		
EG12-3C-2 I		L	L	EG12-34B-6	N		
HF14-1D-2	T		L	EG12-37B-1	N		
HF14-21D-2B		L	L	EG12-3C-1		L	
HF14-27C-7	L		L	EG12-3C-2 II		L	
HF14-30D-2		L	L	EG12-40B-3		ET	
HF14-34B-1B			L	HF14-14B-2	T		
HF14-35B-5C	L		L	HF14-14B-3	T		
HF14-35C-3A			L	HF14-16C-2A	N		
TAP14-145C-2		L	L	HF14-19C-1		T	
TAP14-157B-2			L	HF14-23C-3B		L	
TAP14-190D-3B			L	HF14-23D-2	T		
TAP14-196B-5			L	HF14-24B-3	T	T	
TAP14-203C-21		LT	L	HF14-24C-9A	L	LN	
TAP14-206C-1A			L	HF14-25C-4B		L	
TAP14-206C-5B		L	L	HF14-2D-1	N		
TAP14-208D-1B	L		L	HF14-30A-4A		L	
TAP14-210D-1	L		L	HF14-30C-2C			
TAP14-211D-1			L	HF14-31A-1		T	
TAP14-212D-2	LT	L	L	HF14-39B-7	E	E	
TAP14-213C-1			L	HF14-40D-4	T		
TAP14-216C-5	T	L	L	HF14-43C-2B	N		
EG12-20E-3B			LT	HF14-51C-4	N		
EG12-30B-3B	LT	L	LT	HF14-6A-3	L	LT	
TAP14-147B-4	LT	T	LT	HF14-7C-1	N		
TAP14-202C-1			LT	HF14-7C-5B	N		
TAP14-202C-4		T	LT	HF14-8D-3	L		
TAP14-215B1-1	LT	L	LT	HF14-9A-1		E	
CQ10-27B-1			N	HM13-14B-3	L		
EG12-27C-3			N	HM13-23b-2d	N		
EG12-31A-3B			N	KML12-11MG-C1		E	
EG12-8A-3	N	N	N	KML12-18MG-C2	L		
HF14-23D-3			N	KML12-26MG-E1	T		
HF14-42C-5B	N	N	N	KML12-2MG-F4		T	
CQ10-30A-4	ENT	N	NT	TAP14-142C-4B	L		

CQ10-28B-3		NT	T	TAP14-145C-1		T	
EG10-15A-2	T	T	T	TAP14-147C-5A		T	
EG10-22C-1	LT	T	T	TAP14-156D-1B		T	
EG12-25B-7B			T	TAP14-158B-2B II		E	
EG12-28D-2	NT	LT	T	TAP14-160D-4	L		
EG12-29A-3		T	T	TAP14-165D-1A	L	L	
HF14-14A-1			T	TAP14-166B-1B	L		
HF14-15C-4B			T	TAP14-16C-3		L	
HF14-1D-3	T		T	TAP14-178D-1A		T	
HF14-22C-9	T		T	TAP14-180D-3A	L	L	
HF14-27D-2			T	TAP14-183C-2	T	T	
HF14-28B-2		LT	T	TAP14-183C-5	L		
HF14-32B-4			T	TAP14-197C-5		L	
HF14-35B-5B			T	TAP14-198D-5A II	T	T	
HF14-38D-1			T	TAP14-202C-3	T		
HF14-38D-2			T	TAP14-210B-1		T	
HF14-39B-6		T	T	TAP14-211B-1	L	LT	
HF14-52C-8	L	T	T	TAP14-214D-1B		L	
HF14-8D-1			T	TAP14-220D-1	T	L	
TAP14-111A-4B		T	T	TAP14-224B-1		L	
TAP14-114A-2	T	T	T	TAP14-226B-A	L	L	
TAP14-132B-2		L	T	TAP14-227B-1A	L	L	
TAP14-147D-5B	T		T	TAP14-230C1-1	L		
TAP14-190D-3A	T	T	T	TAP14-3D-2D	L		
				KML12-14MG-B2a	E		

[a]Sorted first by ESKAPE activity, then *Leishmania donovani*, then *Naegleria fowleri* and lastly by *Mycobacterium tuberculosis*. [b]Treatments: Control, no epigenetic regulation treatment; DNMT, treated with DNA methyltransferase inhibitor 5-azacytidine; HDAC, treated with histone deacetylase inhibitor sodium butyrate. [c]Activity: E, ESKAPE pathogen panel (see Table S1); L, *L. donovani* (see Table S3); N, *N. fowleri* (see Table S4); T, *M. tuberculosis* (see Table S2).
